# Investigation of Digital Sun Sensor Technology with an *N*-Shaped Slit Mask

**DOI:** 10.3390/s111009764

**Published:** 2011-10-18

**Authors:** Min-Song Wei, Fei Xing, Bin Li, Zheng You

**Affiliations:** State Key Laboratory of Precision Measurement Technology and Instruments, Department of Precision Instruments and Mechanology, Tsinghua University, Beijing 100084, China; E-Mails: wms09@mails.tsinghua.edu.cn (M.-S.W.); lbin@tsinghua.edu.cn (B.L.); yz-dpi@mail.tsinghua.edu.cn (Z.Y.)

**Keywords:** sun sensor, *N*-shaped slit, linear CCD, simulation, prototype design

## Abstract

Nowadays sun sensors are being more widely used in satellites to determine the sunray orientation, thus development of a new version of sun sensor with lighter mass, lower power consumption and smaller size it of considerable interest. This paper introduces such a novel digital sun sensor, which is composed of a micro-electro-mechanical system (MEMS) mask with an *N*-shaped slit as well as a single linear array charge-coupled device (CCD). The sun sensor can achieve the measurement of two-axis sunray angles according to the three sun spot images on the CCD formed by sun light illumination through the mask. Given the CCD glass layer, an iterative algorithm is established to correct the refraction error. Thus, system resolution, update rate and other characteristics are improved based on the model simulation and system design. The test of sun sensor prototype is carried out on a three-axis rotating platform with a sun simulator. The test results show that the field of view (FOV) is ±60° × ±60° and the accuracy is 0.08 degrees of arc (3σ) in the whole FOV. Since the power consumption of the prototype is only 300 mW and the update rate is 14 Hz, the novel digital sun sensor can be applied broadly in micro/nano-satellites, even pico-satellites.

## 1. Introduction

As an essential component of satellites, the sun sensor has been widely used to measure the incident angle of the sunrays in the satellite-fixed coordinates [1,2]. Sun appearance sensors and analog sun sensors are representative of conventional types [3]. Given the requirements for accuracy, power consumption and size, conventional sun sensors cannot be used any longer in micro/nano-satellites. With the development of imaging devices, such as array Charge-Coupled Devices (CCD) or Active Pixel Sensors (APS), these digital sun sensors have been an attractive focus of research [4,5]. By calculating the sunray angles from the location of sun spots formed on the imaging device, digital sun sensors have the merits of a large field of view as well as high accuracy and reliability, which exactly meet the requirements and trends of sun sensors [6].

Recent years have seen substantial growth in the research on digital sun sensors and related products. Those sensors that adopt planar APS as focal plane detectors usually can determine two-axis sunray angles with higher accuracy and resolution [7–11]. For instance, TNO TPD has a long history of work on digital sun sensors and their product based on the planar APS can achieve an accuracy of 0.024° (2σ) in the whole 120° × 120° FOV, with a power consumption of 1 W [7]. Another two-axis digital sun sensor developed by Tsinghua University also exploited a planar CMOS APS image sensor and its angle accuracy was 0.1° in the whole 128° × 128° FOV, with a power consumption of 2.5 W [9]. On the other hand, those sensors that adopt linear image detectors to achieve two-axis sunray angles have also been studied [12–14]. It has been proven that two linear image sensors mounted perpendicularly function well to determine the sun vector. Such a digital sun sensor with low power consumption (500 mW) was implemented in [12] and its accuracy was 0.05° in FOV of 90°. What is more, Sinclair Interplanetary has designed a series of products from SS256 to SS441 with linear image detectors. The digital sun sensor SS441 realized an accuracy of 0.1° in ±70° FOV and consumed only 300 mW [13]. Nevertheless, its update rate is only 5 Hz, which is mainly caused by the complex algorithm used.

Considering all the aforementioned shortcomings, such as high power consumption [7,9], small FOV [12] and low update rate [13], this article presents a novel digital sun sensor with large FOV, high accuracy, low power consumption, and high update rate which can achieve the measurement of two-axis sunray angles with a single linear CCD. This kind of sun sensor is composed of a micro-electro-mechanical system (MEMS) mask and a single linear CCD. Its field of view is ±60° × ±60° and the accuracy is 0.08 degrees of arc (3σ) in the whole FOV. The prototype only consumes 300 mW and the update rate is 14 Hz.

## 2. Principle and Modeling

### 2.1. Principle of Novel Digital Sun sensor

**Figure 1.**
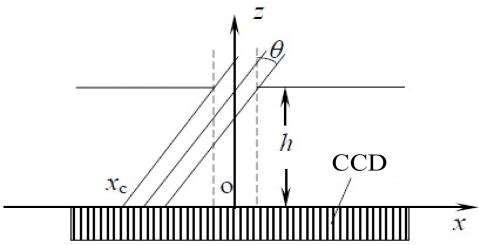
Schematic of digital sun sensor with single slit.

**Figure 2.**
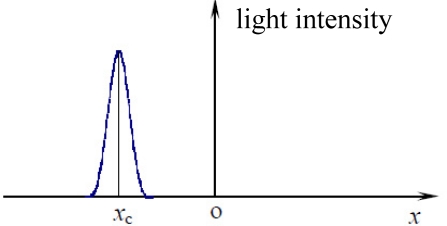
Light intensity distribution of digital sun sensor with single slit.

A linear CCD based sun sensor usually adopts the combination of an optical mask with a single slit on it and a linear CCD detector (see Figure 1) [15]. When the sunray moves toward the sun sensor at a certain angle, the mask plane can block most of the sunray while only a small portion can pass through the slit and reach the detector plane, forming a light spot (see Figure 2).

By analyzing the location of the spot, the sun vector can be reconstructed and the incident angle *θ* can be calculated as:

(1) $$\text{tan}\;\theta =\frac{x_{c}}{h}$$

where *x_c_* denotes the distance between the sun spot and the slit sight point and *h* denotes the distance between the mask plane and the focal plane.

**Figure 3.**
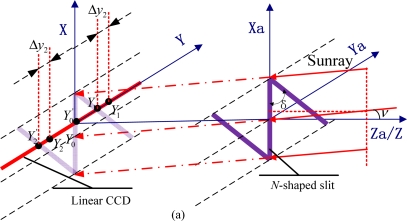

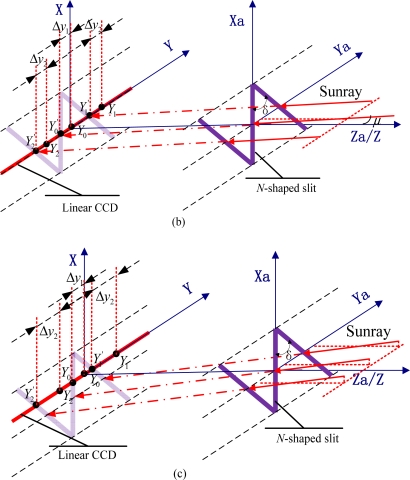
Principle schematic of digital sun sensor with N-shaped slit: **(a)** incident sunray deflects in the Xa-Za plane; **(b)** incident sunray deflects in the Ya-Za plane. **(c)** sunray travels at a random angle of incident.

To maximize the merits of the linear CCD and accomplish the measurement of two-axis sunray angles, a novel scheme has been introduced in this paper. We put forward a mask with an *N*-shaped slit on it to meet the acquirements (see Figure 3).

The special slit is composed of a central slit and two paralleling diagonal slits, which interconnect end to end, like the italic ‘*N*’. And the linear CCD locates just below the mask at a certain distance *h*. Initially, the focal plane parallels to the mask plane while the central slit is perpendicular to the linear CCD.

**Figure 4.**
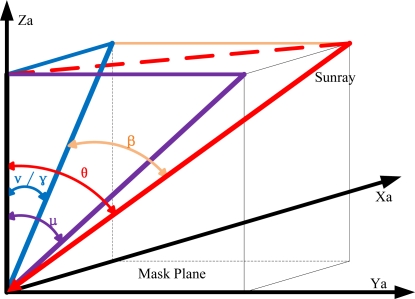
Illustration of angle definitions.

The principle of the novel digital sun sensor is based on the different shifts of the three detected sun spots. It is assumed that three sun spots denoted as *Y*_0_, *Y*_1_ and *Y*_2_ are detected on the default situation (the sunray is parallel to the Z/Za-axis). Compared with the default situation, when an incident sunray deflects in the Xa-Za plane—where *v* denotes the defection angle as shown in Figure 3(a), the central spot remains still while the other two spots shift the same distance in the same direction. When the incident sunray deflects in the Ya-Za plane—where *μ* denotes the defection angle as indicated in Figure 3(b), all three spots shift the same distance in the same direction. Generally, if the sunray travels at a random angle of incident in the FOV, the three spots trend in two distinguishable responses subject to different orientations of the sunray. As shown in Figure 3(c), Δ*y*_1_ which denotes the distance between *Y*_0_ and *Y*_0_′ contains the information related to the deflection angle in the Ya-Za plane while Δ*y*_2_ which denotes the distance between *Y*_1_ and *Y*_1_′ (or between *Y*_2_ and *Y*_2_') contains the information related to the deflection angles both in Ya-Za plane and Xa-Za plane. Taking vector superposition into account, the two-axis sunray angles can be calculated as:

(2) $$\mu =\text{arctan}\;\left(\frac{\Delta y_{1}}{h}\right)=\text{arctan}\;\left(\frac{y_{1m}-{\bar{y}}_{1}}{h}\right)$$

(3) $$\nu =\text{arctan}\;\left(\frac{\Delta y_{2}-\Delta y_{1}}{h\;\text{tan}\;\delta }\right)=\text{arctan}\;\left(\frac{\left(y_{2m}-{\bar{y}}_{2})-(y_{1m}-{\bar{y}}_{1}\right)}{h\;\text{tan}\;\delta }\right)$$

where *μ*, *v* denote the sunray horizontal and azimuth orientation in the satellite-fixed coordinates, respectively, which can be illustrated clearly in Figure 4; *ȳ*_1_ denotes the initial distance between the central sun spot and the origin of coordinates while *ȳ*_2_ denotes the initial distance between either sideways sun spot and the origin of coordinates; *y*_1m_ denotes the measurement distance between the central sun spot and the origin of coordinates while *ȳ*_2_ denotes the measurement distance between either sideways sun spot and the origin of coordinates; and *δ* donates the angle between the central slit and the diagonal slit.

**Figure 5.**
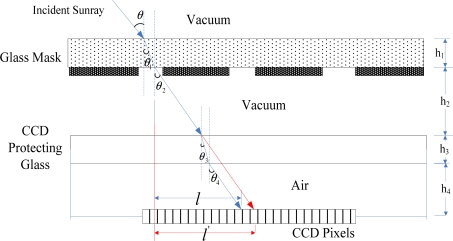
Illustration of the refraction error.

Given the fact that Euler angles are more widely used to scale the attitude of a satellite, we could adapt the equations into the form of Euler angles (*α*, *β*, *γ*) which are defined as mode zxy. When the sunray travels with the incident angle of *θ*, all related angles can be observed in Figure 5, except the angle *α* which cannot be measured by a two-axis sun sensor.

On the basis of the analysis of fundamental geometrical relations, we have:

(4) $$\beta =\text{arctan}\;(\text{tan}\;\mu \;\text{cos}\;\nu )=\text{arctan}\;\left(\frac{\left(y_{1m}-{\bar{y}}_{1}\right)}{\sqrt{{\left[(y_{2m}-{\bar{y}}_{2})-(y_{1m}-{\bar{y}}_{1})\right]}^{2}+h^{2}}}\right)$$

(5) $$\gamma =\nu =\text{arctan}\;\left(\frac{\left(y_{2m}-{\bar{y}}_{2})-(y_{1m}-{\bar{y}}_{1}\right)}{h}\right)$$

### 2.2. Modeling of Refraction Error Correction

Furthermore, the main error of this digital sun sensor is the sunray refraction error caused by the glass protection layer of the CCD [16]. As shown in Figure 5, we introduce a correction coefficient *k* to measure the error, which can be defined as follows:

(6) $$l=h_{2}\;\text{tan}\;\theta +h_{3}\;\text{tan}(\text{arcsin}\frac{n_{1}\;\text{sin}\;\theta }{n_{2}})+h_{4}\;\text{tan}(\text{arcsin}\frac{n_{1}\;\text{sin}\;\theta }{n_{3}})$$

(7) $$l^{\prime }=(h_{2}+h_{3}+h_{4})\;\text{tan}\;\theta$$

(8) $$k=\frac{l^{\prime }}{l}=\frac{(h_{2}+h_{3}+h_{4})\;\text{tan}\;\theta }{h_{2}\;\text{tan}\;\theta +h_{3}\;\text{tan}(\text{arcsin}\frac{n_{1}\;\text{sin}\;\theta }{n_{2}})+h_{4}\;\text{tan}(\text{arcsin}\frac{n_{1}\;\text{sin}\;\theta }{n_{3}})}>1$$

where *n*_1_, *n*_2_, *n*_3_ denote the light refractive index of the vacuum, CCD protecting glass and air, respectively, and the superscript ′ indicates the ideal value without the refraction.

From the explanation about the principle and the geometrical relations, we have:

(9) $$k=\frac{\left(y_{1m}^{\prime }-{\bar{y}}_{1}\right)}{\left(y_{1m}-{\bar{y}}_{1}\right)}=\frac{\left(y_{2m}^{\prime }-{\bar{y}}_{2})-(y_{1m}^{\prime }-{\bar{y}}_{1}\right)}{\left(y_{2m}-{\bar{y}}_{2})-(y_{1m}-{\bar{y}}_{1}\right)}$$

(10) $$l=\sqrt{{\left(y_{1m}-{\bar{y}}_{1}\right)}^{2}+{\left((y_{2m}-{\bar{y}}_{2})-(y_{1m}-{\bar{y}}_{1})\right)}^{2}}$$

So the fundamental equations can be corrected with *k* as follows:

(11) $$\mu =\text{arctan}\;\left(k\frac{y_{1m}-{\bar{y}}_{1}}{h}\right)$$

(12) $$\nu =\gamma =\text{arctan}\;\left(k\frac{\left(y_{2m}-{\bar{y}}_{2})-(y_{1m}-{\bar{y}}_{1}\right)}{h}\right)$$

(13) $$\beta =\text{arctan}\;\left(\frac{k(y_{1m}-{\bar{y}}_{1})}{\sqrt{{\left[(y_{2m}-{\bar{y}}_{2})-(y_{1m}-{\bar{y}}_{1})\right]}^{2}k^{2}+h^{2}}}\right)$$

The key process to correct the refraction error is to calculate the value of *k*, however, it is obvious that *k* is a function of incident angle *θ* while *θ* cannot be measured directly. We have noticed that *l* can be computed directly from Equation (10) according to the measurement of the spots. Although *l* is a function of incident angle *θ* from Equation (6) as well, there is no analytic solution for *θ*. To deal with this problem, an iterative algorithm based on the Newton iteration method has been established. Substitution of *n*_1_ and *n*_3_ with constant 1 into Equation (6) yields the basic iterative equation:

(14) $$(h_{2}+h_{4})\;\text{tan}\;\theta +h_{3}\;\text{tan}(\text{arcsin}\frac{\text{sin}\;\theta }{n_{2}})-l=0$$

Then the incident angle can be calculated through iteration as follows:

(15) $$\theta_{k+1}=\theta_{k}-\frac{F(\theta_{k})}{F^{\prime }(\theta_{k})}$$

where:

(16) $$F(\theta_{k})=(h_{2}+h_{4})\;\text{tan}\;\theta_{k}+h_{3}\;\text{tan}(\text{arcsin}\frac{\text{sin}\;\theta_{k}}{n_{2}})-l$$

(17) $$F^{\prime }(\theta_{k})=\frac{\left(h_{2}+h_{4}\right)}{{\text{cos}}^{2}\;\theta_{k}}+h_{3}\frac{\text{cos}\;\theta_{k}}{{\text{cos}}^{2}\;(\text{arcsin}\frac{\text{sin}\;\theta_{k}}{n_{2}})\sqrt{n_{2}^{2}-{\text{sin}}^{2}\;\theta_{k}}}$$

The initial incident angle for iteration can be obtained through the two-axis angles measured with the equation below:

(18) $${\text{tan}}^{2}\;\theta ={\text{tan}}^{2}\;\nu +{\text{tan}}^{2}\;\mu$$

To sum up, the correction coefficient *k* can be calculated from the sun spots obtained by using the iterative algorithm for incident angle *θ*, and the sun angles (*μ*, *v*) or (*β*, *γ*) can be derived from the Equations (11–13). Furthermore, this algorithm can be applied to both CMOS APS and CCD based sun sensors.

## 3. Simulation Analysis

To optimize the features of the sun sensor, the relationship between the structural parameters and the system functional characteristics should be carefully considered. Therefore, the FOV and system resolution are analyzed in this section for the design of optical head, based on the Equations (2) and (3). These two fundamental equations are analogous, indicating that the incident angle is subject to the spot displacement and the constant quantity *h* or *h*tan*δ*. If we assume that *δ* = 45°, the constant in both equations would be substantially the same, which also results in the same resolution and FOV in both axes. Since we have declared *δ* = 45°, the equation of system resolution based on Equations (2) and (3) can be abstractly presented as:

(19) $$d(y)=\frac{h}{{\text{cos}}^{2}\;(a)}d(a)$$

where *d*(*y*), *d*(*a*) denote the distance resolution of CCD and the angle resolution of the sun sensor at a certain-axis sunray angle of *a*, respectively. The relationship between FOV and *h* can be presented as follows:

(20) $$\text{tan}\;\theta =\frac{\sqrt{\Delta y_{1}^{2}+{\left(\Delta y_{2}-\Delta y_{1}\right)}^{2}}}{h}$$

**Figure 6.**
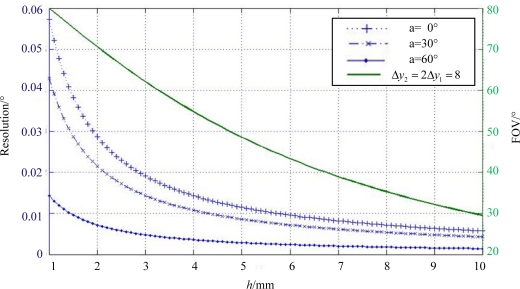
Simulation of resolution and FOV subject to *h.*

From Figure 6, we can conclude that the greater *h* the higher resolution, while on the contrary, the higher *h* the smaller FOV. That is to say, the trade-off between higher system resolution and larger sensor FOV should be considered during the design of *h*. In addition, the resolution varies at different incident angles which can also be revealed from Figure 6, and the greater incident angle the higher resolution.

The first priority of our design is that *h* should satisfy the requirement of FOV, followed by the optimization of the system resolution. According to this criterion, we calculate the relationship between *h* and FOV from Equation (20), when the other parameters are explicit, such as the geometric dimensioning of the CCD. Then the parameters of *N*-shaped slit is taken into account to restrain the geometric position of the sun spots to make sure that all three spots are within the photosensitive region of the detector in the whole FOV. Thus, to accomplish a ±60° × ±60° FOV, *h* = 3.5 mm is calculated. A Toshiba product is used as sun imaging detector with pixel size 8 μm long by 64 μm wide.

**Figure 7.**
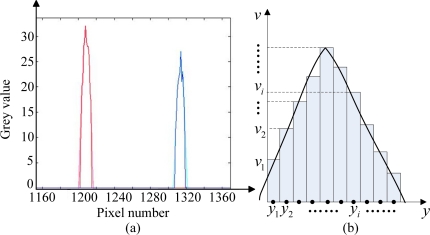
Illustration of the centroiding algorithm. **(a)** data plot of two sun spots detected by CCD; **(b)** abstract of sun spot.

As for the optimization of system resolution, we introduce a first-order centroiding algorithm by having the pixel resolution of CCD subdivided from 1 pixel into 0.1 pixel [17,18]. Since the sun spot detected by the CCD and sampled by the process unit (see Figure 7(a)) actually composes of several serial points with certain grey value, so if we consider one sun spot as an assembly of rectangles as shown in (Figure 7(b)), the centroid of the assembly can indicate the accurate location of the sun spot

From basic physical law, the sum of the serial rectangles’ gravitational potential energy equals the gravitational potential energy of the assembly. If we assume the gravitational potential energy along axis of grey value is zero and the unit area density is 1, then the total energy can be calculated as follows:

(21) $$\sum_{i=1}^{n}\left[(v_{i}\Delta y\right)g\times y_{i}]=[\sum_{i=1}^{n}\left(v_{i}\Delta y\right)]g\times y_{c}$$

where *y_i_*, *y_c_* denote the pixel number of the rectangles within the sun spot and the centroid of the sun spot, respectively, and *v_i_* denotes the grey value of the corresponding point:

(22) $$y_{c}=\frac{\sum_{i=1}^{n}\left(y_{i}\times v_{i}\right)}{\sum_{i=1}^{n}v_{i}}$$

**Figure 8.**
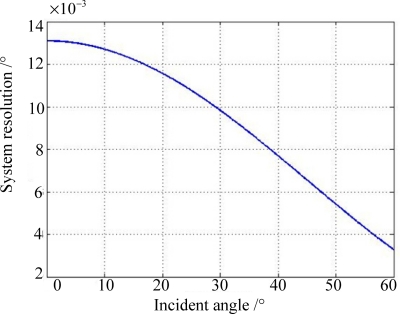
System resolution with respect to the incident angle.

Since the distance resolution of chosen CCD can be subdivided into 0.8 μm, the system resolution can be simulated as shown in Figure 8 from Equation (19). We can conclude that the worst solution occurs at the zero incident angle and the system solution has been designed to be superior to 0.02°.

By employing the Toshiba CCD, which can provide a pixel resolution of 0.8 μm with the help of centroiding algorithm, the FOV is ±60° × ±60°, and system resolution is 0.02° when *h* is designed to be 3.5 mm.

## 4. Mechanical and Electrical Design

**Figure 9.**
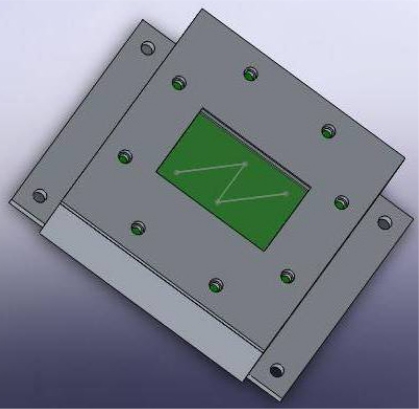
Outline of sun sensor prototype.

To obtain the goals of larger FOV, smaller size and lower power, both hardware and software of the sun sensor must be designed scrupulously. It has been a great help to achieve the aimed FOV by using MEMS-based technology to fabricate the mask. The construction of the sun sensor prototype mainly consists of frames, circuit board and the mask with an *N*-shaped slit (see Figure 9). More accurately, the frames contribute to restrain the distance between the mask plane and the focal plane as well as holding the inner units of the sun sensor; the circuit board integrates the optics to electronics conversion and carries out data processing; the mask forms the image of the incident sunray.

**Figure 10.**
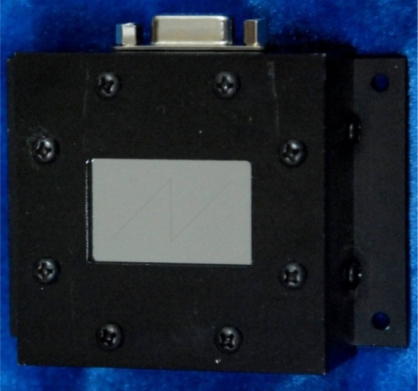
Prototype of digital sun sensor with *N*-shaped slit.

Besides, the mask is fabricated via MEMS processes to archive a thin mask layer for decreasing its adverse effect of the FOV, while it also has the capability to decay the incident sunray to keep the detector working at a proper intensity range [19]. During the brief manufacture process, firstly a Cr mask layer is deposited on the glass base, and then, the photoresist, serving as the lithographic mask, is patterned to form the *N*-shaped slit. After the lithographic process, the *N*-shaped slit will be formed in the Cr mask layer. The prototype is shown in Figure 10.

**Figure 11.**
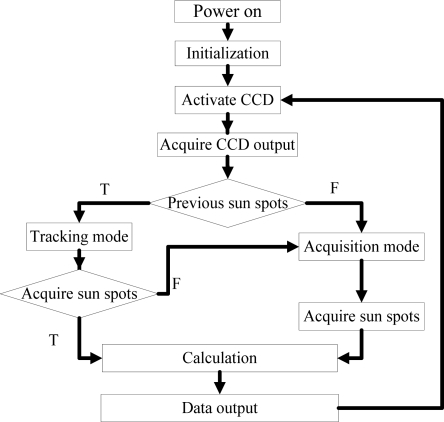
Program flowchart.

The electrical design has been accomplished by integrating all the component devices into one circuit board. And the program is designed as what the program flowchart (see Figure 11) shows. After power-on and initialization of the system, CCD detector will be activated and the analog output of the CCD will be sampled. If there is no previous sun spots data, the whole frame data will be read and analyzed to determine the three sun spots in the acquisition mode. If the sun spots have been detected in the previous cycle, only certain assigned data around the forecasted sun spots will be analyzed to find out the precision sun spots in tracking mode. After the sun spots are acquired based on centroiding algorithm, two-axis sunray angles can be calculated from iterative equations. It is obvious that the tracking mode will save much processing time and then predict a higher update rate.

## 5. Experiments and Results

**Figure 12.**
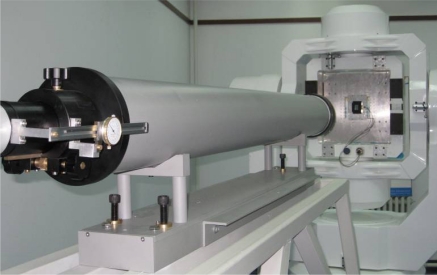
Test system for the sun sensor.

In the test and calibration of the sun sensor, the set-up at ambient conditions serves as the testing system, which consists of a sun simulator and a three-axis gimbals rotary table (see Figure 12).

**Figure 13.**
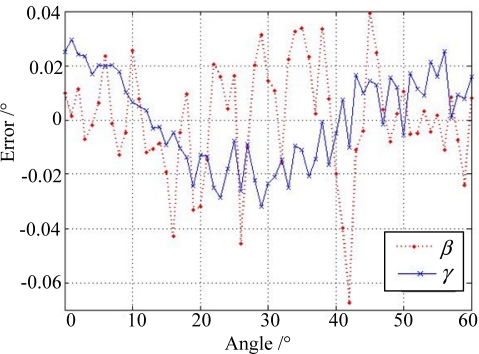
Measurement error statistics of sun sensor performance test.

The sun simulator can send a parallel light beam and the rotating platform can provide different incident sunray angles with a position accuracy of 0.001°. The performance test indicates that the FOV of the sun sensor is larger than ±60° × ±60°, and the maximum error between the measured sun position in the form of incident angle and the setting position through the rotary table is less than 0.08 degrees of arc (see Figure 13 for the half FOV statistics).

The entire performance characteristics as well as physical parameters of the sun sensor have been summarized in Table 1.

## 6. Conclusions

In this paper, a novel digital sun sensor to meet the requirements of the modern application of micro/nano-satellites has been proposed. This sun sensor relies on a MEMS mask with an *N*-shaped slit and a single linear array CCD to achieve two-axis sunray angles by distinguished measurement of the sun spots. Furthermore, a correction coefficient has been introduced with the analysis of the refraction error to improve the accuracy. In addition, system design and simulation to accomplish the prototype of the sun sensor are also presented. The experimental results show that the field of view is ±60° × ±60° and the accuracy is 0.08 degrees of arc (3σ) in the whole FOV. The sun sensor can work at an update rate of 14 Hz, while consuming only 300 mW. The novel sun sensor introduced in this paper provides a broad application in the future, for it accomplishes a high accuracy with light mass, small size and low power consumption.

## Figures and Tables

**Table 1.** Performance of sun sensor.

**Characteristics**	**Performance**

FOV	±60° × ±60°
Accuracy	0.08° (3σ)
Resolution	0.02°
Size	80 mm × 60 mm × 30 mm
Mass	133 g
Power consumption	300 mW
Update rate	14 Hz
